# Gene Copy Number Analysis for Family Data Using Semiparametric Copula Model

**DOI:** 10.4137/bbi.s839

**Published:** 2008-09-26

**Authors:** Ao Yuan, Guanjie Chen, Zhong-Cheng Zhou, George Bonney, Charles Rotimi

**Affiliations:** 1 National Human Genome Center, Howard University, U.S.A; 2 Center for Research on Genomics Global Health, NHGRI, NIH, U.S.A; 3 SuiZhou Central Hospital, SuiZhou, HuBei, 441300 P.R. China

**Keywords:** cluster, copula, family data, gene copy number, semiparametric model

## Abstract

Gene copy number changes are common characteristics of many genetic disorders. A new technology, array comparative genomic hybridization (a-CGH), is widely used today to screen for gains and losses in cancers and other genetic diseases with high resolution at the genome level or for specific chromosomal region. Statistical methods for analyzing such a-CGH data have been developed. However, most of the existing methods are for unrelated individual data and the results from them provide explanation for horizontal variations in copy number changes. It is potentially meaningful to develop a statistical method that will allow for the analysis of family data to investigate the vertical kinship effects as well. Here we consider a semiparametric model based on clustering method in which the marginal distributions are estimated nonparametrically, and the familial dependence structure is modeled by copula. The model is illustrated and evaluated using simulated data. Our results show that the proposed method is more robust than the commonly used multivariate normal model. Finally, we demonstrated the utility of our method using a real dataset.

## Introduction

The gene copy number (also called “copy number variants”—CNV) is the number of copies of a particular gene in the genome of an organism. Recent evidences show that gene copy number (GCN) amplifications and deletions are common characteristics of many genetic diseases. For example, GCN can be elevated in cancer cells as demonstrated in the epidermal growth factor receptor (EGFR) gene in patients with non-small cell lung cancer (Cappuzzo et al. 2005) and also higher copy number of CCL3L1 has been associated with susceptibility to human HIV infection (Gonzalez et al. 2005). Thus identifying these genetic gains and losses provides useful information about specific disease susceptibility or resistance. GCN analysis among normal people within the human genome is also of interest. However, these genetic characteristics are usually not directly observable. Recent technological development in array comparative genomic hybridization (a-CGH) provides scientists with an efficient tool to conduct whole genome and high-density region specific investigation of GCN (Solinas et al. 1997; Pinkel et al. 1998; Snijders et al. 2001).

Briefly, a-CGH technique involves the labeling of genomic DNA from disease tissues (e.g. cancer) and normal control tissue (reference) with different colors (fluorochrome). These samples are then co-hybridize to a metaphase spread from a normal reference cell. After hybridization, emission from each of the two fluorescent dyes is measured, and the signal intensity ratios are indicative of the relative copy number of the two samples. The ratio of the two fluorochrome intensities is then calculated and regions where the disease DNA are amplified or deleted are readily detected on the metaphase spread. The resulting data are in the form of microarrays. This technique not only gives us information about copy number gains and losses in the disease genomic DNA but also allows the identification of the specific chromosomes and the regions of the chromosomes where these changes occurred.

However, the a-CGH data does not provide direct measurements of the GCN changes. Hence, several statistical approaches for analyzing and describing results from these experiments have been developed. Differences exist in these approaches and newer approaches addressing some of the limitations of existing method are needed. For example, some of these methods do not take into account the spatial dependence within the chromosome (Hodgson et al. 2001; Pollack et al. 2002; Cheng et al. 2003; Wang et al. 2004) while others have implemented such dependence structure into their models to enhance the inference (Jong et al. 2004; Picard et al. 2004; Fridlyand et al. 2004; Eilers et al. 2004). With the exception of a few that are Bayesian (Barash and Friedman, 2002; Daruwala et al. 2004; Broet and Richardson, 2005), most of the existing methods are frequentist’s. All of the existing methods are designed for analyzing data from unrelated persons and are therefore effective in explaining horizontal changes in GCN. However and when available, family data present wonderful opportunity to investigate the vertical kinship effects of GCN as well as the horizontal changes. For this type of data, the main challenge is to model the high dimensional familial dependence structure, and no such approach was found following a careful review of the literature. In this paper, we present such a method in which we used the nonparametric approach to model the marginal distributions and then linked the joint distribution by a copula structure.

Typically, GCN changes observed from a-CGH experiments are classified into three groups corresponding to the three statuses of copy number changes—amplification, deletion and normal; Thus, allowing the microarray responses to have similar features. The practical challenge in the problem that we describe here is that of high dimensionality due to familial dependence among pedigree members. As we indicated above, several of the statistical tools for microarry data clustering deal with low dimensional data (usually one dimensional) and do not take into account the familial dependence among the pedigree members. Such methods can be divided into two main groups, the model based and non-model based (semiparametric). The former assumes specific probability models for the sub-distributions of response in each cluster and is efficient when the specifications are correct but may be seriously biased if implemented specifications deviate from the true unknown models. The semiparametric does not make any assumption about the models except that of a mixing structure, in which the unknown sub-distributions are estimated nonparametrically from the data themselves, and the inference is robust. This method is adequate when the data size is large so that the sub-distributions can be estimated accurately. Yuan and He (2008) proposed such a method for low dimensional microarray clustering for independent data generated from unrelated persons. For data with high dimensionality, the commonly used multivariate normal model rarely fits the actual data, and the nonparametric method is not directly applicable in cluster analysis, so neither of the above models based or non-model based methods are suitable for analyzing the dependence and high dimensionality of family data.

In statistics, the copula is a widely used tool for modeling the dependence structure of high dimensional data (Sklar, 1959; Joe, 1997), and is particularly suitable for pedigree data modeling. Here we propose and implement a semiparametric copula model to address this problem. Specifically, the marginal distributions are estimated nonparametrically, the within pedigree dependence structure is modeled by copula with parameters specified by the kinship coefficients. A penalty term is used against non-unimodality of the sub-distributions. The optimal partition is performed using a classification-estimation (of densities)-maximization-estimation (of parameters) algorithm. The algorithm shares the property of ascending the penalized semiparametric likelihood, just like the well known EM algorithm for ascending the parametric likelihood, and thus, under fair conditions, converges to the optimal partition of the microarray.

## The Method

In a-CGH data, the fluorescence ratios between two samples, case and control, are measured across a genomic region. For loci with different copy number changes, the corresponding log-ratio measurement tend to be different. Thus in a-CGH data analysis, often a three-state mixture model is used: deletion state, normal state and amplification state, and we arbitrarily lable them as state 1, 2 and 3. Genes with copy number deletion tend to have smaller log-ratio measurements, those with normal status tend to have moderate measurements, and those with amplification tend to have larger measurements.

We focus on the case of a given chromosome. When there are more than one chromosome under consideration, the method is similar by modeling the chromosomes one by one. Suppose there are *n* loci of interest and *r* pedigrees of individuals. The measurement at each locus for each individual is observed. The *j*-th pedigree has *s_j_* individuals (*j* = 1, …, *r*), at locus *k*, the *l*-th individual of the *j*-th pedigree has microarray measurement *y_jkl_*. Denote *y_jk_* (*y_jk_*_1_,…, *y*_*jks_j_*_)′ be the measurements of the *j*-th pedigree at locus *k*, for each fixed pair (*j*, *k*) the *y_jkl_*’s are familiarly dependent due to kinship.

Generally this question is formulated as a cluster problem, in which each of the gene locus in classified into one of the clusters *B*_1_, *B*_2_ and *B*_3_ represent the three states deletion, normal and amplification. Let *y* be a general random vector of the observation y*_jk_*’s, a mixture model on y is specified as

(1) $$f(y)=\sum_{i=1}^{3}\alpha_{i}f(y|B_{i}),$$

Where *f* (·|*B_i_*) is the sub-density of the responses in the *i*-th cluster, and the *α_i_*’s are the mixing proportions satisfying 0 ≤ *α_i_* ≤ 1, ∑*_i_*_=1_*^k^* *α* = 1. In the literature usually the *f*(·|*B_i_*)’s are specified as multi dimensional normal density functions with cluster specific mean vectors and variance matrices. Typically for this type of pedigree data the dimension is around 10 to 15.

In practice, such high dimensional dependent data hardly conforms to a multivariate normal distribution. A commonly used statistical tool to deal with high dimensional dependence structure is the copula. In this method, it is only necessary to specify each of the marginal densities, and then use a link (copula) to compose all the marginal densities into a joint multivariate density with desired dependence structure. There are large number of copulas to be used, and some optimality criteria to select the best copula for a given problem and data. When the copula is selected, we can incorporate the kinship coefficients among the pedigree members into its dependence structure. Also, there is the question of how to specify the marginal densities. There are various parametric densities to choose from, but if the wrong one is used the results may be seriously biased. On the other hand, for data with large sample size, the nonparametric density can adapt to any distributional feature. Since we do not know the true sub-densities we model each of the marginal densities by nonparametric method for robustness. Finally, a modified BIC criterion is used to select the optimal number of clusters. We describe each of the above steps in different sub-sections below.

### The marginal distributions

Since commonly available pedigree data usually consist of three generations and to account for the age and gender difference, the distributions of the measurements are divided into six groups in the following order: first generation male, first generation female, second generation male, second generation female, third generation male and third generation female. We use *G_s_* to denote the *s*-th group. For example if an individual with observation *y_jkl_* is a second generation female in any given pedigree, she is in group 4, we simply denote *y_jkl_* ∈ *G*_4_, and so on. Denote *f_s_*(·|*B_i_*) be the sub-density of array cluster *i* of group *s*.

Since the *f_s_*(·|*B_i_*)s are unknown, they can be estimated by the well known nonparametric estimator (Rosenblatt, 1969)

(2) $${\hat{f}}_{s}(y_{jkl}|B_{i})=\frac{1}{n_{is}h_{n_{is}}}\sum_{y_{uvw}\in C_{i}\cap G_{s}}K\left(\frac{y_{uvw}-y_{jkl}}{h_{n_{is}}}\right),$$

where *n_is_* is the sample size (number of individuals) of group s in cluster *i*, *K*(·) is arbitrary given kernel density, and *h*_*n_is_*_ is a given bandwidth to be specified below.

In the density estimation literature, the choice of kernel is not of particular importance (Diggle, 1983; Silverman, 1986). Studies suggest that most unimodal densities perform about the same as the other when used as a kernel, and the choice between kernels can be made on other grounds such as computational efficiency. However, there are some popular options in practice for different reasons. For some general introduction for the choice of kernels, we refer to Silverman (1986) and Scott and Wand (1991). The normal kernel (i.e. *K*(·) is the density function of the standard normal distribution) is widely used in practice for convenience and other nice features.

In contrast, the choice of bandwidth is crucial in density estimation (Silverman, 1986). Interesting proposals which address this problem can be found in Fan and Gijbels (1992). There is literature on automatic methods that attempt to minimize a lack-of-fit criterion, such as integrated squared error. From Silverman (1986), we choose to use the bandwidth

(3) $$h_{n_{is}}=0.9{\hat{\sigma }}_{is}{\left(n_{is}\right)}^{-1/5}$$

where *σ̂_is_*^2^ is the empirical variance of the *y_jkl_*’s in the *s*-th group and the *i*-th cluster.

In the copula formulation we also need the corresponding marginal distribution functions. Let *Fs*(·|*B_i_*) denote the marginal distribution functions for cluster *i* and group *s*, *F̂_s_*(·|*B_i_*) for its empirical estimate,

(4) $${\hat{F}}_{s}(y_{jkl}|B_{i})=\frac{1}{n_{is}}\sum_{y_{uvw}\in C_{i}\cap G_{s}}\chi \left(y_{uvw}\leq y_{jkl}\right),$$

where χ(·) is the indicator function.

### The joint distribution

The copula is a commonly used statistical tool to model multivariate joint distribution, it appeared in the early work of Hoeffding, Fréchet and others and formally introduced by Sklar (1959). We first give a very brief account of it and we refer to Hunchinson and Lai (1990); Joe (1997) and Nelson (1999) for detailed review.

A function *C* defined on [0, 1]*^d^* is a *d*-variate copula if *C*(*F*_1_(*x*_1_), …, *F_d_* (*x_d_*)) is a joint distribution function for any marginal distribution functions *F*_1_(*x*_1_), …, *F_d_* (*x_d_*). The marginal distributions of *C*(*F*_1_(*x*_1_), …, *F_d_* (*x_d_*)) itself are just *F*_1_(*x*_1_), …, *F_d_* (*x_d_*). This property provides a convenient way to construct a joint distribution with given marginal ones. On the other hand, given a set of marginal distribution functions *F*_1_(*x*_1_), …, *F_d_* (*x_d_*), there is a unique copula *C* such that *C*(*F*_1_(*x*_1_), …, *F_d_* (*x_d_*)) is the true joint distribution of them (Sklar, 1959). Also, for any joint *d*-dimensional distribution function *F*(…), let *F_i_*^−1^(·) be the quantile functions of the *i*-th margin, then the function *C*(*x*_1_,…, *x_d_*) = *F*(*F*_1_^−1^(*x*_1_),…, *F_d_*^−1^(*x_d_*) is a *d*-variate copula. Let *c* (…) be the density function (the total derivative) of *C*(…) when exists. Let *f_i_* (·) be the density function of *F_i_*(·), the density function *f* (*x*_1_, …, *x_d_*) of the copula distribution function *C*(*F*_1_(*x*_1_), …, *F_d_* (*x_d_*)) is given by

(5) $$f(x_{1},\ldots ,x_{d})=c(F_{1}(x_{1}),\ldots ,F_{d}(x_{d}))\prod_{i=1}^{d}f_{i}(x_{i}).$$

Given a copula, the dependence structure can be characterized in several ways, including Pear-son’s correlation, Kendall’s tau, Spearman’s rho, tail dependence, etc. Kendall’s tau is generally easier to compute for copulas, so we use this dependence measure. For a two-dimensional copula, Kendall’s tau is given by

$$\begin{array}{l} \tau =4\int_{0}^{1}\int_{0}^{1}C(u,\upsilon )c(u,\upsilon )dud\upsilon -1\\ =2P((X_{1}-X)(Y_{1}-Y)>0)-1, \end{array}$$

where (*X*_1_, *Y*_1_) and (*X*, *Y*) are independent with the same distribution. −1 ≤ *τ* ≤ 1, *τ* = 0 for independence, −1 and 1 for perfect negative and positive dependence. Genest et al. (1995) suggested a pseudo-likelihood approach to estimate the dependence parameters, in which the observed data is transformed via the empirical marginal distributions to obtain pseudo-data that are used in the estimation. Using the special relationships among relative pairs, we can implement the dependence parameters in the copula via the relationships among kinship coefficients, Kendall’s tau and the copula dependence parameters without estimation.

For pedigree data, the dependence relationships among familial members (*i*, *j*) are best described by the kinship coeffcients, *γ_ij_* = Δ_7_*_ij_*/2 + Δ_8_*_ij_*/4, where Δ_7_*_ij_*, Δ_8_*_ij_*, Δ_9_*_ij_* are the condensed kinship coefficient (Jacquard, 1974) between relative pair *i* and *j*. The Δ*_kij_s* (*k* = 1, …, 9) are the probabilities for the nine possible condensed identical by descent (IBD) status as in Jacquard (1974), in which Δ_7_*_ij_*, Δ_8_*_ij_* and Δ_9_*_ij_* are commonly used in practice. They are the population probabilities of sharing 2, 1 and 0 genes IBD for relative pair (*i*, *j*), without regard to their particular genotypes, but only (*i*, *j*)’s kinship relationships, under the Mendelian inheritance. Also, 2*γ_ij_* is the expected proportion of gene IBD for relative pair(*i*, *j*) at this locus. For convenience we list the values of these coefficients for some common relative pairs (Lange, 1997), and we compute corresponding Kendall’s tau in the last column after the computations below.

For trait underlined by single locus or multiple loci, Pearson’s correlation for relative pair (*i*, *j*) is 2*γ_ij_* (Lange, 1997). Assume that gene copy number change statuses are determined only by the underlying genetic sources, and that the amounts of dependence among them are additive with respect to their shared genetic sources. Then at any fixed locus, Kendall’s tau between a fixed type of relative pair (*i*, *j*) is (Appendix)

(6) $$\tau_{ij}=2\Delta_{7ij}+(3/2)\Delta_{8ij}+\Delta_{9ij}-1.$$

As is true for Pearson’s correlation, we postulate that Kendall’s tau remain the same, or approximately so, when the trait is influenced by multiple loci. As the kinship coefficients are already known as in Table 1, we get Kendall’s tau by the above relationships and in turn, the dependence parameters in the copula model is obtained from the relationship among the dependence parameters and kendal’s tau for specified copula. Thus we can easily implement the dependence kinship coefficients in the copula in terms of Kendall’s tau without estimating them. For this, we first need to review several commonly used copulas. Note, for family data the dependence are not constant among different relative types, hence copulas with constant dependence parameters, such as Clayton’s copula or Frank’s copula can not be used here.

### Multivariate normal copula

Let Φ*_d_* (·, Θ) be the *d*-variate normal distribution function with mean vector **0** and correlation matrix Θ = (*θ_ij_*), φ*_d_* (·, Θ) be its density function. Φ(·) be the one-dimensional standard normal distribution function, and Φ^−1^(·) be its quantile (inverse) function. The multivariate normal copula is defined as

$$\begin{array}{l} C(u_{1},\ldots ,u_{d};\Theta )=\Phi_{d}(\Phi^{-1}(u_{1}),\ldots ,\Phi^{-1}(u_{d});\Theta ),\\ (u_{1},\ldots ,u_{d})\in {\left[0,1\right]}^{d} \end{array}$$

with density

$$\begin{array}{l} c(u_{1},\ldots ,u_{d};\Theta )=\varphi_{d}(\Phi^{-1}(u_{1}),\ldots ,\Phi^{-1}(u_{d});\Theta )\\ \prod_{j=1}^{d}\left(1/\varphi (\Phi^{-1}(u_{j})\right). \end{array}$$

Thus for given marginal distribution functions *F*_1_(·), …, *F_d_* (·) and their densities *f*_1_(·), …, *f_d_* (·), the joint distribution function for the multivariate normal copula with these given margins is

$$\begin{array}{l} F(x_{1},\ldots ,x_{d})=C(F_{1}(x_{1}),\ldots ,F_{d}(x_{d});\Theta )\\ =\Phi_{d}(\Phi^{-1}(F_{1}(x_{1})),\ldots ,\\ \Phi^{-1}(F_{d}(x_{d}));\Theta ) \end{array}$$

with density

$$\begin{array}{l} f(x_{1},\ldots ,x_{d})=c(x_{1},\ldots ,x_{d})\\ =\varphi_{d}(\Phi^{-1}(F_{1}(x_{1})),\ldots ,\Phi^{-1}(F_{d}(x_{d})))\\ \prod_{j=1}^{d}\frac{f_{j}(x_{j})}{\varphi (\Phi^{-1}(F_{j}(x_{j}))}. \end{array}$$

For the distribution in (6), any lower dimensional joint distributions have the same form. For example the (*i*, *j*)-th joint distribution function is *F*(*x_i_*, *x_j_*) = Φ_2_(Φ^−1^(*F_i_*(*x_i_*)), Φ^−1^(*F_i_*(*x_j_*)); Θ*_ij_*), where Θ*_ij_* is the (*i*, *j*) sub-block of the matrix Θ. From Table 1 and in this case, Spearman’s and Kendall’s tau are the same. For this copula, Spearman’s rho (Kendall’s tau) and the dependence parameters θ*_ij_*’s in normal copula are related by (Lindskog, 2000)

(7) $$\tau_{ij}=\frac{6}{\pi }\text{arcsin}\frac{\theta_{ij}}{2},\;\;\;or\;\;\;\theta_{ij}=2\text{sin}(\tau_{ij}\pi /6).$$

By relationships (6) and (7), the dependence parameters θ*_ij_*’s in the multivariate normal copula are easily obtained given the τ*_ij_*’s, which are computed via the known kinship coefficients Δ*_kij_*’s, as long as we know the kin type of relative pair (*i*, *j*).

### Multivariate T-copula

Let Θ be the correlation matrix given in the multivariate normal distribution, *x* = (*x*_1_, …, *x_d_*)’ The density function *d*-dimensional *T*-distribution with *r* degrees of freedom is

$$\begin{array}{l} q_{r}(x_{1},\ldots ,x_{d})=\frac{\Gamma ((r+d)/2)}{{\left(r\pi \right)}^{d/2}\Gamma (r/2)|\Theta {|}^{1/2}}\\ {\left(1+\frac{1}{r}x^{\prime }\Theta^{-1}x\right)}^{-(r+d)/2}. \end{array}$$

The corresponding copula density is

$$\begin{array}{l} c(u_{1},\ldots ,u_{d})=q_{r}(Q_{r}^{-1}(u_{1}),\ldots ,Q_{r}^{-1}(u_{d}))\\ \prod_{j=1}^{d}\left(1/q_{r}(Q_{r}^{-1}(u_{j}))\right), \end{array}$$

where *Q_r_*(·) is the distribution function of the *T*-distribution with *r* degrees of freedom, and *q_r_*(·) is its density function. Given marginal distribution functions *F*_1_(·), …, *F_d_*(·) and their densities *f*_1_(·), …, *f_d_*(·), the joint density with the copula defined by this multivariate *T*-distribution is

$$\begin{array}{l} f(x_{1},\ldots ,x_{d})=q_{r}(Q_{r}^{-1}(F_{1}(x_{1})),\ldots ,Q_{r}^{-1}(F_{d}(x_{d})))\\ \prod_{j=1}^{d}\frac{f_{j}(x_{i})}{q_{r}(Q_{r}^{-1}(F_{j}(x_{j})))}. \end{array}$$

For this copula, the relationships between the θ*_ij_*’*s* and the τ*_ij_*’*s* are the same as for the multivariate normal copula.

### Selection of copula

Given several candidate copulas *C*_1_, …, *C_h_* with densities *c*_1_, …, *c_h_*, a natural question is how to select the optimal copula for the data. Let *F̂_jl_*(·) be the estimated marginal distribution for individual *l* in pedigree *j* (although there are only six different versions of them). For example, if individual (*j*, *l*) is in group *s*, then *F̂_jl_*(·) *F̂_s_*(·). The *F̂_s_*(·)’s are defined as

$${\hat{F}}_{s}(y_{jkl})=\frac{1}{n_{s}}\sum_{yu\upsilon w\in G_{s}}\chi (y_{uvw}\leq y_{jkl}),$$

where *n_s_* is the number of observations in group *s*.

When there are parameters to be estimated in the copula, the optimal copula can be selected by AIC criteria (Oakes, 1989; Dias and Embrechts, 2003). Here, our copula has no parameters to be estimated, by the likelihood principle and (5), an intuitive way is to select the *C* with

$$C=\text{arg}\;\underset{i}{\text{max}}\prod_{j=1}^{r}\prod_{k=1}^{n}c_{i}({\hat{F}}_{j1}(y_{\mathrm{jk}1}),\ldots ,{\hat{F}}_{\mathrm{jsj}}(y_{\mathrm{jksj}}))$$

or equivalently, to avoid computation overflow or underflow,

(8) $$\begin{array}{l} C=\text{arg}\;\underset{i}{\text{max}}\frac{1}{r}\prod_{j=1}^{r}\prod_{k=1}^{n}\text{log}\;c_{i}({\hat{F}}_{j1}(y_{\mathrm{jk}1}),\ldots \\ {\hat{F}}_{\mathrm{jsj}}(y_{\mathrm{kk}s_{j}})). \end{array}$$

This is equivalent to choosing the copula with the largest likelihood.

Now for given copula, the joint density for the data *y* = {*y_jkl_*} is modeled by

(9) $$\hat{f}(y)=\prod_{j=1}^{r}\prod_{k=1}^{n}\prod_{i=1}^{3}\alpha_{i}\hat{f}(y_{jk}|B_{i})$$

where

$$\begin{array}{l} \hat{f}(y_{jk}|B_{i})=c({\hat{F}}_{j1}(y_{jk1}|B_{i}),\ldots ,{\hat{F}}_{{js}_{j}}(y_{{jks}_{j}}|B_{i}))\\ \prod_{l=1}^{s_{j}}{\hat{f}}_{jl}(y_{jkl}|B_{i}). \end{array}$$

We point out that although we used the same notation *c*, for different families, the number of individuals may differ and so are the dimensionalities of the *c*’s.

However, under the semi-parametric mixture model assumption, the sub-distributions can take any shape, even the shape of the entire distribution, and as a result any cluster partition will give about the same likelihood value via (9). So optimizing (9) over all possible cluster partitions will not be able to identify the desired clusters. Thus we put some constraints on the selection of clusters such that the sub-distribution is approximately unimodal and optimizing model (9) will give the desired clusters, as in Yuan and He (2008). The reference Yuan and He will be refered to as YH in subsequent citations. However there are two major differences between the method we are proposing and that of YH. Our method can handle high-dimensional data and the link among the marginal densities in copula.

Specifically, let *g*(·|*B_i_*) be the multivariate normal density with mean given by the sample mean for data in *B_i_*, and covariance matrix Θ, for observations in *B_i_* (*i* = 1, …, 3), and denote *g* = (*g*_1_, *g*_2_, *g*_3_), where *g_i_* = *g*(·|*B_i_*). *g* is used as shape constraints for the *f̂* (·|*B_i_*)*s*. Intuitively, for each fixed *i*, when the ‘correct’ partitions are specified, the differences between the *f̂* (·|*B_i_*)*s* and *g*(·|*B_i_*)s will be relatively small. The Kullback-Leibler divergence ***D f̂*** **(*B*** **i)**, ***g*** **(*B****_i_***)** is be used to quantify this difference between the two densities *f̂* (·*|B_i_*) and *g*(·*|B_i_*) with ***D f̂*(*Bi*)**, ***g*** **(*Bi*)) =∫*Bif̂***(***y|Bi***) log[***f̂***(***y|Bi***)/*g*(*y* |*B_i_*)]*dy*. Note that ***D*(*f̂*(*Bi*))**, ***g*** **(*Bi*)** is non-negative and is zero only if *f̂*(·|*B*) ≡ *g*(·|*B_i_*). An empirical version of it is given by

$$D(\hat{f}(B_{i}),g(B_{i}))=\sum_{y_{\mathrm{jk}}\in B_{i}}\text{log}[\hat{f}(y_{\mathrm{jk}})/g(y_{\mathrm{jk}})]$$

and we set

$$D(\hat{f},g|B):=\sum_{i=1}^{3}D(\hat{f}(B_{i}),g(B_{i})).$$

Let ***L***_0_ (*α* | ***y, f̂ B***) be the log-likelihood of (9). Now, instead of optimizing (9), we optimize over all possible partitions of clusters, the penalized log-likelihood,

(10) $$\begin{array}{l} L(\alpha |y,\hat{f},g,B)=L_{0}(\alpha |y,\hat{f},B)-\lambda D(\hat{f},g|B)\\ =\sum_{j=1}^{r}\sum_{k=1}^{n}\text{log}\left(\sum_{i=1}^{3}\alpha_{i}{\hat{f}}^{1-\lambda }(y_{\mathrm{jk}}|B_{i})g^{\lambda }(y_{\mathrm{jk}}|B_{i})\right), \end{array}$$

for some 0 ≤ λ ≤ 1 to be specified. This model can be viewed as an extension of the traditional mixture model. When λ = 0, it corresponds to a nonparametric specification of sub-distributions, when λ = 1 it is a full parametric model given by the *g*(·|*B_i_*)s, and when 0 < λ < 1 it corresponds to an intermediate model. By doing this, we are forcing the distributions to be close to normal, more than what is needed for unimodal. The tunning parameter λ is chosen according to simulation for the given type of data. The choice of a multi-variate normal here is for convenience as other choices could be made but may result in additional complication.

### The CEME algorithm

However, directly optimizing the mixture model (10) is usually not easy. A common practice of estimating the cluster membership of each observation in the data while evaluating the maximum likelihood estimate *α̂* of α in (10) is the EM algorithm (Dempster et al. 1977). The EM algorithm is a much easier (though much slower) endeavor computationally than the direct optimization.

For fixed *k*, let *u_ij_* = 1 if the *i*-th locus belongs to the *j*-th cluster, *u_i_* = (*u_i_*_1_, *u_i_*_2_, *u_i_*_3_) be its membership vector, and *u* = {*u_ij_*}. Treating *u* as missing data, (*y*, *u*) is referred to as the “complete” data. Then the likelihood for the “complete” data is

$$\prod_{j=1}^{r}\prod_{k=1}^{n}\prod_{s=1}^{3}{\left(\alpha_{s}\hat{f}(y_{jk}|B_{s})\right)}^{u_{ks}}.$$

Although we used the same notation *f̂*(*y_jk_*|*B_s_*) for each fixed *B_s_*, the dimension of the data y*_jk_* may vary for different pedigree *j*, as well as the density *f̂*(*y_jk_*|*B_s_*). The corresponding log-likelihood is

$$\begin{array}{l} L_{0}(\alpha |y,\hat{f},u,B)=\sum_{j=1}^{r}\sum_{k=1}^{n}\sum_{s=1}^{3}u_{\mathrm{ks}}(\text{log}\alpha_{s}\\ +\text{log}\hat{f}(y_{\mathrm{jk}}|B_{s})). \end{array}$$

By the same reason as (10), we optimize the penalized “complete data” log-likelihood

(11) $$\begin{array}{l} L(\alpha |y,\hat{f},q,u,B)=\sum_{j=1}^{r}\sum_{k=1}^{n}\sum_{s=1}^{3}u_{\mathrm{ks}}(\text{log}\alpha_{s}\\ +\text{log}[{\hat{f}}^{1-\lambda }(y_{\mathrm{jk}}|B_{s})\\ g^{\lambda }(y_{\mathrm{jk}}|B_{s})]), \end{array}$$

where *g*(*y_jk_* | *B_s_*) is the analogue of *f̂* (*y_jk_*|*B_s_*). The above log-likelihood is optimized iteratively, with the clusters *B_s_*’s are classified along each iteration. We specify the starting values at iteration zero as below.

### Starting values

Set *α*_s_^(0)^ = 1/3, (*s* = 1 2 3,); *u_ks_*^(0)^ = 1/3, (*s* = 1 2 3); *k* = 1, …, *n*). Divide the *n* loci into 3 region of roughly equal sizes, and lable them as the *B_s_*^(0)^’s. Let *f̂*^(0)^ (·|*B_s_*^(0)^) be the nonparametric estimate of *f_j_*(·) using only the measure responses in *B_s_*^(0)^. Denote (*B*^(^*^t^*^)^ = *B*_1_^(^*^t^*^)^, *B*_2_^(^*^t^*^)^, *B*_3_^(^*^t^*^)^) be the estimate of *B* = (*B*_1_, *B*_2_, *B*_3_) at the *t*-th iteration of the algorithm.

Given the current *t*-th iteration estimates *α*^(^*^t^*^)^ = *α*_1_^(^*^t^*^)^, *α*_2_^(^*^t^*^)^, *α*_3_^(^*^t^*^)^), *u_ij_*^(^*^t^*^)^’*s B* (*t*), *f* (t)(·*B_s_*^(^*^t^*^)^)’s and *q*^(^*^t^*^)^ (·|*B_s_*^(^*^t^*^)^)’s from the *t*-th iteration, we update them in the (*t* + 1)-th iteration according to the following CEME steps.

1. *Classification-step*: Each response locus *k*, is classified into a candidate cluster *B̃_s_*, if
  $$\begin{array}{l} \prod_{j=1}^{r}\left(\left(\alpha_{s}^{\left(t\right)}f^{\left(t\right)}({\text{y}}_{jk}|B_{s}^{\left(t\right)}\right)\right)\\ =\underset{1\leq l\leq 3}{\text{max}}\prod_{j=1}^{r}\left(\left(\alpha_{l}^{\left(t\right)}f^{\left(t\right)}({\text{y}}_{jk}|B_{s}^{\left(t\right)}\right)\right). \end{array}$$
  This is the optimal classification rule in the sense of minimizing the expected loss (Anderson, 1984), and it is also the so-called Bayesian assignment. In the cases of ties, we use uniform random assignment among the tied clusters. Let *B̃* = (*B̃*_1_, *B̃*_2_, *B̃*_3_) be a candidate classification of the clusters after this step.
2. *Expectation-step*: Let *U_ks_*’s be the associated random variables of the *u_ks_*’s, and *g*^(^*^t^*^)^ (·|*B_s_*^(^*^t^*^)^) be the multi-dimensional normal density with mean and covariance matrix empirically estimated from the data in *B_s_*^(^*^t^*^)^(*s* = 1 2 3).
3. As in YH, for *k* = 1, …, n; *s* = 1, 2, 3, we have
  $$\begin{array}{l} {\hat{u}}_{\mathrm{ks}}^{\left(t+1\right)}:=E\left(U_{\mathrm{ks}}|y,\alpha^{\left(t\right)},f^{\left(t\right)},g^{\left(t\right)}\right)\\ =\frac{\Pi_{j=1}^{r}\left(a_{s}^{\left(t\right)}f^{{\left(t\right)}^{1-\lambda }}(y_{\mathrm{jk}}|B_{s}^{\left(t\right)})g^{{\left(r\right)}^{\lambda }}(y_{\mathrm{jk}}|B_{s}^{\left(t\right)})\right)}{\Sigma_{l=1}^{3}\Pi_{j=1}^{r}\left(a_{l}^{\left(r\right)}f^{{\left(t\right)}^{1-\lambda }}(y_{\mathrm{jk}}|B_{l}^{\left(t\right)})g^{(t)\lambda }(y_{\mathrm{jk}}|B_{l}^{\left(t\right)})\right)}, \end{array}$$
  where the expectation is taken with respect to the constrained log-likelihood *L*. Denote *u*^(^*^t^*^+1)^ = {*u_ks_*^(^*^t^*^+1)^}.
4. *Maximization-step*: Compute the MLE *α^(t^* ^+ 1)^ of a given *u*^(^*^t^* ^+ 1)^ as in YH
  $$\alpha_{s}^{\left(t+1\right)}=\frac{\Sigma_{k=1}^{n}u_{ks}^{\left(t\right)}}{\Sigma_{k=1}^{n}\Sigma_{k=1}^{3}u_{kl}^{\left(t\right)}}=\frac{1}{n}\sum_{k=1}^{n}u_{ks}^{\left(t\right)}.$$
5. *Estimation-step*: To update the estimation of the density ***f*** ^(^*^t^*^)^(·) of current iteration *t* to ***f*** ^(^*^t^*^+1)^(·) for the next iteration *t* + 1, we first compute candidate sub-marginal density ***f̃****_jl_*(·*|B̃_s_*) for individual *l* in pedigree *j* at locus *k* and cluster *s*. If this individual is in group *v*, then
  $${\tilde{f}}_{jl}(y_{jkl}|{\tilde{B}}_{s})=\frac{1}{{\tilde{n}}_{s\upsilon }{\tilde{h}}_{s\upsilon }}\sum_{y_{abc}\in {\tilde{B}}_{s}\cap G_{\upsilon }}K\left(\frac{y_{abc}-y_{jkl}}{{\tilde{h}}_{s\upsilon }}\right)$$
  with
  $$\begin{array}{l} {\tilde{h}}_{s\upsilon }=0.9{\tilde{\sigma }}_{s\upsilon }{\left({\tilde{n}}_{sv}\right)}^{-1/5},(j=1,\ldots ,r;l=1,\ldots ,s_{j};\\ k=1,\ldots ,n;s=1,2,3;\upsilon =1,\ldots ,6) \end{array}$$
  where *ñ_sv_* is the number of responses for group *v* in cluster *B̃_s_*, and *σ̃_sv_*^2^ is sample variance of this group. Similarly, the candidate sub-marginal distribution functions are
  $$\begin{array}{l} {\tilde{F}}_{jl}(y_{jkl}|{\tilde{B}}_{s})=\frac{1}{{\tilde{n}}_{s\upsilon }}\sum_{y_{abc}\in {\tilde{B}}_{s}\cap G_{\upsilon }}\chi (y_{abc}\leq y_{jkl}),\\ (j=1,\ldots ,r;l=1,\ldots ,s_{j};k=1,\ldots ,n;\\ s=1,2,3;\upsilon =1,\ldots ,6). \end{array}$$

Then use (5) to get the candidate sub-joint density for the *j*-th pedigree at locus *k*, as follows

$$\begin{array}{l} \tilde{f}(y_{jk}|{\tilde{B}}_{s})=c({\tilde{F}}_{j1}(y_{j1k}),\ldots ,{\tilde{F}}_{js_{j}}(y_{js_{j}}k))\prod_{l=1}^{j}{\tilde{f}}_{jl}(y_{jkl}),\\ \left(j=1,\ldots ,r;k=1,\ldots ,n;s=1,2,3\right) \end{array}$$

and that for all the pedigree at locus *k* is

$$\prod_{j=1}^{r}\tilde{f}(y_{jk}|{\tilde{B}}_{s}),(k=1,\ldots ,n;s=1,2,3).$$

Let *g̃* be the reference densities corresponding to *B̃*.

We update the quadruple (*B*^(^*^t^* ^+ 1)^, *f*^(^*^t^* ^+ 1)^, *F*^(^*^t^* ^+ 1)^, *g*^(^*^t^* ^+ 1)^) as

$$\begin{array}{l} (B^{(t+1),}f^{\left(t+1\right)},F^{(t+1),}g^{\left(t+1\right)})=\{\begin{array}{l} (\tilde{B},\tilde{f},\tilde{F},\tilde{g}),\\ \left(B^{\left(t\right)},f^{\left(t\right)},F^{\left(t\right)},g^{\left(t\right)}\right) \end{array}\\ \{\begin{array}{l} \text{if }L(\alpha^{\left(t+1\right)}|y,\tilde{f},\tilde{g},\tilde{B})\geq L(\alpha^{\left(t\right)}|y,f^{\left(t\right)},g^{\left(t\right)},B^{\left(t\right)}),\\ \text{otherwise}. \end{array} \end{array}$$

The estimate of *f* (·) at the (*t* + 1)-th iteration is then

$${\hat{f}}^{\left(t+1\right)}(\cdot )=\sum_{s=1}^{3}{\hat{\alpha }}_{s}^{\left(t+1\right)}{\hat{f}}^{\left(t+1\right)}(\cdot |B_{s}^{\left(t+1\right)}).$$

Note that at each iteration *t*, α^(^*^t^*^)^, *B*^(^*^t^*^)^, and the *u_ij_*^(^*^t^*^)^s are updated, but not necessarily so for *f*^(^*^t^*^)^ and *g*^(^*^t^*^)^.

The above four steps are iterated until convergence of (*α*^(^*^t^*^)^, *f*^(^*^t^*^)^, *B*^(^*^t^*^)^). (Note by the following Proposition, we may check the stability of the *α*^(^*^t^*^)^ as a simple criterion for the convergence of the triple). We may use the relative error criterion for the convergence of the (*α*^(^*^t^*^)^’s, that is, for some pre-specified *δ* > 0, we stop the iteration when ∑*_s_*_=1_^3^|(*α_s_*^(^*^t^*^+1)^ − *α_s_*^(^*^t^*^)^)/*α_s_*^(^*^t^*^)^|≤ *δ*. Typically, *δ* ≤ 0.01.

As in YH, we have

#### Proposition

*For each fixed k, the sequence* {*L*(*α*^(^*^t^*^)^|*y*, *f* ^(t)^, *g*^(t)^, *B*^(t)^)} *is increasing in t, and there is a stationary point* (α^*^, *f* ^*^, B^*^) *satisfying*

$$\begin{array}{l} \prod_{j=1}^{r}\left(\alpha_{s}^{*}f^{*}(y_{jk}|B_{s}^{*})\right)=\underset{l}{\text{max}}\prod_{j=1}^{r}\left(\alpha_{l}^{*}f^{*}(y_{jk}|B_{l}^{*})\right),\\ \forall k\in B_{s}^{*}(s=1,2,3). \end{array}$$

*When* (α^*^, *f* ^*^, B^*^) *is the unique stationary point, we have, as t* → ∞,

$$(\alpha^{\left(t\right)},f^{\left(t\right)},B^{\left(t\right)})\to (a^{*},f^{*},B^{*}).$$

## Application

### Simulation study

We simulate *r* = 10 pedigrees, each has four individuals, father, mother and two sibs, and we assume there are *n* = 200 loci of interest, which are divided into 3 clusters as *B*_1_ = (1, 80), *B*_2_ = (81, 150) and *B*_3_ = (151, 200), with cluster means *μ*_1_ = (4.9, 4.2), *μ*_2_ = (9.9, 9.2) and *μ*_3_ = (14.9, 14.2) for male and female individuals. We generated two datasets by simulation using the normal copula and multi-normal models. Each of the datasets were analyzed using both normal copula and multi-normal models.

To simulate data from the Multivariate normal copula model, let *A* be the Cholesky decomposition of Θ. To sample from this copula distribution: for *k* = 1, …, *n* and *i* = 1, …, 5

1. generate *r* independent samples ***Z***_1_*_k_*, …, ***Z****_rk_* from *N* (**0**, *I*_4_).
2. Let ***u****_lk_* = *A****Z****_lk_* (*l* = 1, …, *r*).
3. For *k*∈*B_i_*, if *j* is for male, set *x_ljk_* = Φ(*u_l_*_1_*_k_*) + *μ_k_*_1_; if *j* is for female, set *x_ljk_* = Φ(*u_l_*_1_*_k_*) + *μ_k_*_2_, and *x_lk_* = (*x_l_*_1_*_k_*, …, *x_l_*_4_*_k)_*, where Φ(·) is the distribution function of the standard normal. Then *x*_1_*_k_*, …, *x_rk_* is a sample from the 4-variate normal copula model with correlation matrix Θ. The results are displayed in Table 2 below, with tuning parameter *λ* of values 0.25, 0.5, 0.75 and 1.

In the above, *λ* = 1 corresponds to a normal model, and 0 < *λ* <1 correspond to a mixed model. For this type of data, the model has difficulty in parameter convergence for small values of *λ*, reflecting the fact that the multivariate data distribution is too noisy for nonparametric part of the model to work alone; thus a parametric unimodal component is needed to help cluster the data. The normal copula model has larger likelihood value in all these cases. This means the normal copula model is more robust than the multivariate normal model. For the data from multi-normal model, when *λ* = 0.5, 38 loci from cluster three are classified to cluster two. Over all, *λ* = 0.75 performs well for all the data set, and so we recommend this value of *λ* in this analysis.

To assess the robustness of the method, we simulated larger data sets with family sizes of 4 and 5, each with 100 families and 200 candidate loci. The simulated clusters and means for male and female are: cluster one, 1–70, (4.9, 4.2); cluster two, 81–150, (8.9,8.2) and cluster three, 171–200, (12.9,12.2) respectively. To reflect some complexity we added minor clusters to some of the clusters. The means for male and female for the minor clusters are 71–80, (5.4, 4.7) and 151–170, (12.4,11.7). The results are summarized in Table 3.

Overall, the results are consistent: the smaller the value of λ, the better the model fitness, as indicated by larger likelihood value. This means that the non-parametric model component capture the data distribution in fine details. But in many cases, the computation breaks down for *λ* = 0 as pointed out earlier. It is seen that for either the data generated from multi-normal or normal copula distributions, the overall performances of the semiparametric model is robust for a range of the tuning parameter *λ*.

### Real data analysis

We use the proposed method to analyze the Genetics Analysis Workshop 15 (GAW15) data set with 14 pedigrees of CEPH Utah families, each with three generations and about a dozen normal individuals. Expression level of genes in lymphoblastoid cells of the above subjects were obtained using the Affymetrix Human Focus Arrays that contain probes for 8,500 transcripts. Gene copy number variations in normal people within human genome has been the subject of study (Freeman et al. 2006; Pugh et al. 2008). For 3,554 of the 8,500 SNPs tested, Morley et al. (2004) found greater variation among individuals than between replicate determinations on the same individual. These 3,554 expression phenotypes (expressed genes) were chosen for copy number change analysis. The first step is to find out the best copula model for the data. We considered three different models, the multi-normal model, the semi-parametric multivariate normal-copula model, and the semi-parametric multivariate T-copula model. Then the criterion in (8) is used to select the optimal model. The average copula likelihood values for the three models are −3217389.15, −2094272.97, −2296408.96 respectively. Thus the semi-parametric multivariate normal-copula model is the best of the three and was used for clustering. The outcome of the analysis of the GAW15 data is displayed in the figure below. The horizontal axis represents the sequential numbering of genes from 1 to 3550, and the vertical axis indicates the classified states of the genes with 1, 2 and 3 representing deletion, normal and amplification.

As shown in the figure, most of the SNPs are in clusters 1 and 3, this observation is consistent with the large variation of the expression levels. The SNPs with deletion status are more likely to be contained in cluster 1, and those with amplification status are more likely to be in cluster 3.

## Concluding Remarks

We proposed, studied and demonstrated a semipa-rametric copula method for microarray-SNP genomewide association analysis using pedigree data. We successfully implemented the kinship relationship into the model for more robust analysis of family data than the commonly used multivariate normal model.

## Figures and Tables

**Figure 1:**
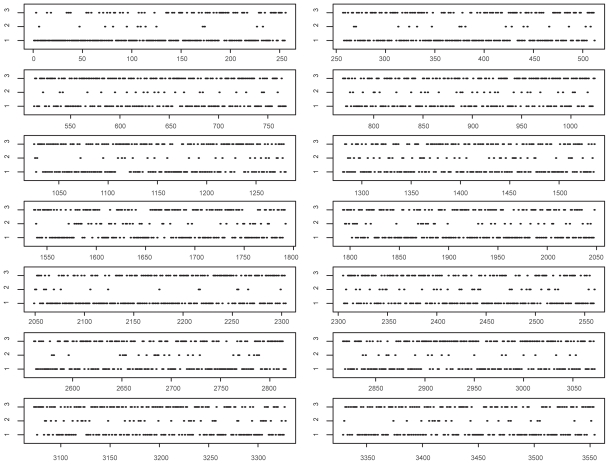


**Table 1:** Kinship coeffcient for selected relative pairs.

Relationship	Δ_7_	Δ_8_	Δ_9_	γ	τ
Grand parent-offspring	0	1/2	1/2	1/8	1/4
Parent-Offspring	0	1	0	1/4	1/2
Half Siblings	0	1/2	1/2	1/8	1/4
Full Siblings	1/4	1/2	1/4	1/4	1/2
First Cousins	0	1/4	3/4	1/16	1/8
Double First Cousins	1/16	6/16	9/16	1/8	1/4
Second Cousins	0	1/16	15/16	1/64	1/32
Uncle-Nephew	0	1/2	1/2	1/8	1/4

**Table 2:** Cluster results for normal copula and multi-normal models for 10 pedigrees and 200 loci.

Data	λ	Cluster 1	Cluster 2	Cluster 3	Log-likelihood
Normal Copula	0.25	1–80	81–150	151–200	−11438825.76
	0.50	1–80	81–150	151–200	−22088970.51
	0.75	1–80	81–150	151–200	−32749199.84
	1.00	1–80	81–150	151–200	−43412062.82
Multi-normal Model	0.50	1–80	81–150(+38)	151–200(−38)	−3117275.81
	0.75	1–80	81–150	151–200	−3644388.69
	1.00	1–80	81–150	151–200	−4652713.94

**Table 3:** Summary cluster results from normal copula and multi-normal data sets for 100 pedigrees with 4 or 5 Family Members.

Pedigree size	Data	λ	Cluster 1	Cluster 2	Cluster 3	Log-likelihood
4	Normal Copula	0.25	1–80	81–150	151–200	−5503651.81
		0.50	1–80	81–150	151–200	−10207757.93
		0.75	1–80	81–150	151–200	−14924762.04
		1.00	1–80	81–150	151–200	−19645406.52
4	Multi-normal Model	0.50	1–80	81–150(+9)	151–200(−9)	−1716690.87
		0.75	1–80	81–150	151–200	−2213287.79
		1.00	1–80	81–150	151–200	−2726810.52
5	Normal Copula	0.25	1–80(−7)	81–150(+7)	151–200	−7841636.12
		0.50	1–80(−7)	81–150(+7)	151–200	−15364258.65
		0.75	1–80(−7)	81–150(+7)	151–200	−22940970.23
		1.00	1–80(−6)	81–150(+6)	151–200	−28643031.41
5	Multi-normal Model	0.25	1–80	81–150	151–200	−1639830.86
		0.50	1–80	81–150	151–200	−2250676.24
		0.75	1–80	81–150	151–200	−2874104.71
		1.00	1–80	81–150	151–200	−3503763.43

## Appendix

Proof of (6): Let (*X*_1_, *Y*_1_) be the traits of relative pair (*i*, *j*); (*X*, *Y*) be an independent copy of (*X*_1_, *Y*_1_); and *A**_l_* be the event that a relative pair share l alleles IBD (l = 0, 1, 2). Given a relative pair (*i*, *j*), by definition *P*(*A*_2_) = Δ _7_*_ij_*, *P*(*A*_1_) = Δ_8_*_ij_*, and *P*(*A*_0_) = Δ_9_*_ij_*. By the assumption that GCN change is determined by the underlying genetic source and given *A*_2_, the pair (*i*, *j*) share the same genetic source at the locus and the same copy number change status; thus *X*_1_ = *Y*_1_, *X* = *Y*. Note that the random variables *X*_1_ and *X* are of continuous type and *P*((*X*_1_ − *X*) (*Y*_1_ − *Y*). 0|*A*_2_) = *P*((*X*_1_ − *X*)^2^. 0|*A*_2_) = 1. Also, *X*_1_ − *X* and *Y*_1_ − *Y* are random variables symmetric around 0, thus given *A*_0_, *X*_1_ − *X* and *Y*_1_ − *Y* are independent, so the events (*X*_1_ − *X*)(*Y*_1_ − *Y*). 0 and (*X*_1_ − *X*)(*Y*_1_ − *Y*), 0 are completely random, each with probability 1/2, i.e. *P*((*X*_1_ − *X*)(*Y*_1_ − *Y*)|*A*_0_) = 1/2. By the additivity assumption, given *A*_1_, the probability of the event (*X*_1_ − *X*)(*Y*_1_ − *Y*). 0 is the average of those for cases of given *A*_2_ and *A*_0_, i.e. *P*((*X*_1_ − *X*)(*Y*_1_ − *Y*). 0|*A*_1_) = 3/4. Then at any fixed locus, Kendall’s tau between a fixed type of relative pair (*i*, *j*) is

$$\begin{array}{l} \tau_{ij}=2P((X_{1}-X)(Y_{1}-Y)>0|A_{2})P(A_{2})\\ +2P((X_{1}-X)(Y_{1}-Y)>0|A_{1})P(A_{1})\\ +2P((X_{1}-X)(Y_{1}-Y)>0|A_{0})P(A_{0})P(A_{0})-1\\ =2\times 1\times \Delta_{7ij}+2\times (3/4)\times \Delta_{8ij}+2\times (1/2)\times \Delta_{9ij}-1\\ =2\Delta_{7ij}+(3/2)\Delta_{8ij}+\Delta_{9ij}-1. \end{array}$$
